# Association of DNA Repair Gene APE1 Asp148Glu Polymorphism with Breast Cancer Risk

**DOI:** 10.1155/2015/869512

**Published:** 2015-07-16

**Authors:** Fatima AlMutairi, Akbar Ali Khan Pathan, Mohammed Alanazi, Manal Shalaby, Huda A. Alabdulkarim, Abdullah Alamri, Abdulrahman Al Naeem, Moammad Elrobh, Jilani P. Shaik, Wajahatullah Khan, Zahid Khan, Narasimha Reddy Parine

**Affiliations:** ^1^Genome Research Chair, Department of Biochemistry, College of Science, King Saud University, Riyadh 11451, Saudi Arabia; ^2^Integrated Gulf Biosystems, Riyadh 11391, Saudi Arabia; ^3^Genetic Engineering and Biotechnology Research Institute (GEBRI), City for Scientific Research and Technology Applications, Alexandria 21934, Egypt; ^4^The Comprehensive Cancer Center, King Fahad Medical City, Riyadh 11525, Saudi Arabia; ^5^Department of Women's Imaging, King Fahad Medical City, Riyadh 11525, Saudi Arabia; ^6^Department of Basic Sciences, College of Science and Health Professions, King Saud Bin Abdul Aziz University for Health Sciences, Riyadh 11426, Saudi Arabia

## Abstract

*Objective*. The aim of this study was to investigate the role of APE1 Asp148Glu polymorphism in breast cancer progression in Saudi population. *Methods*. We examined the genetic variations (rs1130409) in the DNA base excision repair gene APE1 at codon 148 (Asp148Glu) and its association with breast cancer risk using genotypic assays and *in silico* structural as well as functional predictions. *In silico* structural analysis was performed with Asp148Glu allele and compared with the predicted native protein structure. The wild and mutant 3D structures of APE1 were compared and analyzed using solvent accessibility models for protein stability confirmation. *Results*. Genotypic analysis of APE1 (rs1130409) showed statistically significant association of Asp148Glu with elevated susceptibility to breast cancer. The *in silico* analysis results indicated that the nsSNP Asp148Glu may cause changes in the protein structure and is associated with breast cancer risk. *Conclusion*. Taken together, this is the first report that established that Asp148Glu variant has structural and functional effect on the APE1 and may play an important role in breast cancer progression in Saudi population.

## 1. Introduction

The incidence of breast cancer is highest among other cancers and is the main cause of cancer related deaths in Saudi women [1]. Compared to developed and Western region nations, the age adjusted rate for breast cancer in Saudi population is 4-5-fold lower; however, the median age of diagnosis is 49 years, which is significantly lower compared to Western patients [2, 3]. There is significant evidence that inadequate repair of DNA damage plays a major role in the progression of cancer and other human diseases [4]. Base excision repair (BER) is involved in repair of oxidative free radical induced DNA lesions. Efficiency of (BER) is suggested to be a major determinant of breast cancer risk [5]. It is a key repair pathway that is accountable for conserving genome stability and consequently protecting from cancer and other diseases by repairing numerous lesions and strand breaks of DNA which are uninterruptedly caused by endogenous and exogenous mutagens [6]. When a single base is damaged, the BER pathway enzymes are responsible for recognizing and repairing the damaged base [7]. The first step in base excision repair pathway uses DNA glycosylases, to remove the damaged base to form an abasic or AP site by cleaving the N-glycosyl bond between the sugar and the base. Following the removal of the damaged base, apurinic/apyrimidinic endonuclease 1 (APE1) hydrolytically cleaves the phosphodiester backbone 5′ to the AP site, resulting in the formation of a 5′-deoxyribose phosphate (5′-dRP) and a 3′-OH primer [8]. At this juncture, DNA polymerase then inserts a correct nucleotide and DNA ligases seal the repaired DNA strand. APE1 is a multifunctional enzyme which is located on chromosome 14q11.2–q12 [9]. It exhibits DNA repair activity and has a role in the reductive activation of many transcription factors. These two functions are encoded by two different sites of APE1 enzyme. The N-terminal region encodes the redox function and the C-terminal region encodes the repair function [10]. The DNA repair activity includes 3′-phosphodiesterase, 3′-phosphatase, and 3-5′-exonuclease activities [11]. To coordinate BER pathway APE1 interacts with PARP1, XRCC1, DNA polymerase *β*, and flap endonuclease 1 (FEN1) [12]. APE1 stimulates all these proteins individually. DNA polymerase then inserts a correct nucleotide and DNA ligases seal the repaired DNA strand. Mutations in this highly regulated mechanism can be caused by single nucleotide polymorphisms (SNPs), which will result in insufficient DNA repair which may enhance DNA lesions [13, 14]. Mutations in APE1 gene may affect its function. If AP sites are not processed, mutagenesis and cellular cytotoxicity can result from blocked DNA replication machinery or from misincorporation of bases opposite the AP site [12, 15]. The APE1 polymorphism at codon 148 has been previously reported to be associated with prostate cancer risk [14]. However, previous reports on the association between APE1 polymorphisms and cancer risk have provided inconsistent results [16–19]. Data pertaining the association of the APE1 polymorphisms with breast cancer risk are also inconsistent [4, 20]. To the best of our knowledge, there is no published report on the association between APE1 SNP variant Asp148Glu (rs1130409) and breast cancer in Saudi population. Thus, this is the first study that investigates APE1 SNP Asp148Glu association with breast cancers using TaqMan assay in Saudi women. Additionally,* in silico* analyses were performed to determine the structural and functional consequence of Glu instead of Asp at codon 148 of APE1 protein.

## 2. Materials and Methods

### 2.1. Study Population

This study was a case-control study that included 100 Saudi female patients diagnosed with breast cancer at King Fahad Medical City Hospital, Riyadh, Saudi Arabia, along with 100 controls. Controls were age-matched and confirmed to be free of cancer and other diseases following thorough physical examinations. The demographic data of each patient and control were recorded (Table 1). Patients and controls were enrolled under King Khalid University Hospital Institutional Review Board approved protocol with written informed consent.

### 2.2. Genotyping

Genomic DNA was extracted from the blood samples of breast cancer cases and controls using QIAmp DNA blood Mini Kit (Qiagen, Valencia, CA) following the manufacturer's instructions. SNP rs1130409 in APE1 gene was genotyped using TaqMan allelic discrimination assay as described previously [21, 22]. Ten percent of the samples were subjected to repeated analysis for verification of genotyping procedures.

### 2.3. Modeling of Mutant Structure

X-ray diffraction structure of APE1 (2O3H) from PDB database was used as a reference to compare the wild-type (Asp148) protein structure with the predicted mutant structure (148Glu) and its solvent accessibility including secondary structures was modeled using molecular dynamics (MD) simulation. The best homology model for APE1 protein with Asp148Glu was selected using I-TASSER server [23]. The mutant APE1 Asp148Glu 3-D structure was predicted using Modeller 9v10 [24]. The predicted model for mutant type protein was evaluated using ProSA-web [25].

### 2.4. Analyzing the Effects of Mutation on Protein Stability

Stability of the mutant protein is checked using prediction of Prediction of Protein Mutant Stability Changes (PoPMuSiC) [26]. The results were based on the selected ΔΔ*G* values in kcal/mol of the predicted APE1 Asp148Glu structure to evaluate the change in folding free energy after mutation (ΔΔ*G*). Additionally, CUPSAT (Cologne University Protein Stability Analysis Tool) was also used to confirm the results [27].

### 2.5. The Effect of Mutant Residue 148Glu on APE1 Structure and Function

The presence of glutamate instead of aspartate at codon 148 of APE1 may affect the overall structure with a potential to alter its activity. The effect of Asp148Glu mutation was analyzed using Have yOur Protein Explained (HOPE) program [28] as described earlier by Alanazi et al. [29].

### 2.6. Molecular Dynamics Simulation

The effect of glutamate at codon 148 on APE1 was studied by comparing with three-dimensional structure of APE1 protein present in the protein databank [PDB: 2O3H]. Structures deduced for APE1 harboring mutation Asp148Glu which is identified to be risk in Saudi breast cancer samples were utilized for the analyses. Biopolymer module in InsightII was used to substitute aspartate with glutamate residue at codon 148 from the fragment library and to add hydrogen atoms to both wild-type and mutated protein structures at pH 7.0 (Accelrys Inc., San Diego, CA). The molecular simulation program CHARMM was used to derive the force fields of both structures [30]. A sequence of energy minimization steps were performed as described by Alanazi et al. [31] on native and mutant protein structures by using InsightII/Discover (Accelrys Inc., San Diego, CA). Following energy minimization, protein structures were analyzed using Discovery Studio 2.5 (Accelrys Inc., San Diego, CA).

### 2.7. Statistical Analysis

Genotype and allelic frequencies were compared using Fisher's exact test (two-tailed) as described by Alanazi et al. [21] to estimate the *χ* ^2^ test and odds ratios (OR) and 95% confidence intervals (CI) to know the variation between cancer cases and controls. All statistical analyses were performed using Statistical Package for the Social Sciences version 21.0 (SPSS Inc., Chicago, IL). The allele and genotype frequencies of APE1 (rs1130409) polymorphisms in the central region population of Saudi Arabia (CRS) were compared with some of the populations of the HapMap database, for example, Utah residents with northern and western European ancestry from the CEPH collection (CEU), Han Chinese in Beijing, China (CHB), Yoruba in Ibadan, Nigeria (YRI), Maasai in Kinyawa, Kenya (MKK), Japanese in Tokyo, Japan (JPT), Gujarati Indians in Houston, Texas (GIH), and Toscans in Italy (TSI) as described previously by Alanazi et al. [32]. Pairwise Chi-square (*χ* ^2^) tests were performed between the central region population of Saudi Arabia (CRS) and other populations using the allele frequencies in a 2 × 2 contingency table to study if the central region of Saudi population (CRS) shows significant differences compared to other populations.

## 3. Results

The present study examined the SNP rs1130409 (Asp148Glu) of APE1 gene in a total of 200 subjects. The distribution of the three genotypes as Asp/Asp, Asp/Glu, and Glu/Glu at codon 148 of the APE1 was significantly different between the controls and breast cancer patients (*χ* ^2^ = 9.44, df = 2, and *p* = 0.0089). The genotype frequencies in breast cancer cases were 0.12, 0.45, and 0.43 for Asp/Asp, Asp/Glu, and Glu/Glu, respectively, whereas in healthy controls the frequencies of Asp/Asp, Asp/Glu, and Glu/Glu were 0.27, 0.46, and 0.27, respectively (Table 2). The heterozygotes (Asp/Glu) and homozygote variant (Glu/Glu) showed significantly higher risk in breast cancer patients compared with the controls (Asp/Glu: OR = 2.20, *χ* ^2^ = 3.87, and *p* = 0.0491; Glu/Glu: OR: 3.58, *χ* ^2^ = 9.42, and *p* = 0.0021). The Glu allelic frequency of rs1130409 was higher (0.655) in the breast cancer patients than that in the control group (0.50) (OR = 1.89, *χ* ^2^ = 9.85, and *p* = 0.0017) (Table 2).

### 3.1. Effect of Age on the Association of APE1 SNP Asp148Glu with Breast Cancer

To examine the association of the SNPs with the age at the time of breast cancer diagnosis, we stratified the patients according to the median age at diagnosis (Table 1) as ≤48 (*n* = 47) or >48 (*n* = 53) years and compared them with age-matched controls. The analyses showed that the SNP rs1130409 did not have any association with breast cancers arising in women at or below 48 years of age (Table 3). However,* APE1* codon 148 variant showed significant association with elderly breast cancer patients (>48 years) with Glu/Glu genotype as well as Glu allele posing higher risk (Table 3).

### 3.2. Effect of ER Status on the Association of APE1 SNP Asp148Glu with Breast Cancer

The association of breast cancer risk with the SNP variant rs1130409 based on the estrogen receptor (ER) status of the cancer patients was also investigated. The genotype distribution in the ER+ (*n* = 55) and ER− (*n* = 45) was independently compared with the genotypes in the control samples (*n* = 100) (Table 4).* APE1* variant rs1130409 showed higher risk in both ER+ and ER− breast cancer patients for Glu/Glu genotype and Glu alleles (Table 4).

### 3.3. Genotype and Allele Frequencies of* APE1* Variant rs1130409 in Saudis and Other Populations

We compared the genotypic and allelic frequencies of the* APE1* SNP rs1130409 in a normal healthy Saudi population with some other populations available in the HapMap database. The observed Asp/Asp, Asp/Glu, and Glu/Glu genotype frequencies in the Saudi population were 0.27, 0.46, and 0.27, respectively (Table 5). Both the Asp (wild-type) and the Glu (variant) allele frequencies were similar and found to be 0.50 in Saudi population. The variant allele frequency ranged from 0.48 in CEU to 0.72 in GIH population. The CRS population frequencies differed significantly from GIH, YRI, and MKK populations based on the pairwise Chi-square (*χ* ^2^) tests (Table 5).

### 3.4. Effect of APE1 Asp148Glu Substitution on Protein Stability

Thermodynamic protein stability variations due to the replacement of Asp148 with 148Glu in the APE1 were predicted using PoPMusic. It revealed that the amino acid alteration resulted in low levels of folding free energy (ΔΔ*G* = 1.16 kcal/mol) and caused structural destabilizing effects. Similar results were observed with CUPSAT as well. APE1 Asp148Glu exhibited unfavorable changes in torsion angles which influenced the overall stability of the protein. APE1 148Glu tertiary structure revealed significant variations due to protein folding in the mutated region between predicted and measured stability changes.

### 3.5. Effect of Mutant Residue 148Glu on APE1 Structure and Function

The mutant Glu residue at position 148 of APE1 protein was bigger than the wild-type Asp residue. Have yOur Protein Explained (HOPE) which collects and combines information from several webservers and databases was used to analyze the effect of Asp148Glu on APE1.

*Domains.* Asp148Glu residue is part of APE1 interpro domain named “Exodeoxyribonuclease III xth” (IPR004808; see http://www.ebi.ac.uk/interpro/entry/IPR004808;jsessionid=BEC787876CD492333C5F42D24F5AFD6B). This domain is annotated with the following Gene-Ontology (GO) terms to indicate its function: nuclease activity (GO: 0004518) and DNA repair (GO: 0006281; see http://www.ebi.ac.uk/QuickGO/GTerm?id=GO:0006281). More broadly speaking, these Gene-Ontology annotations indicate that the domain has a function in hydrolase activity (GO: 0016787; see http://www.ebi.ac.uk/QuickGO/GTerm?id=GO:0016787). This residue is part of an interpro domain named “Endonuclease/exonuclease/phosphatase” (IPR005135; see http://www.ebi.ac.uk/interpro/entry/IPR005135;jsessionid=05F95A5D842EBA0A1048B8D753BB9705). Since the mutant residue was located in a domain which plays an important role primarily in the protein activity, it might disturb this function.

*Conservation.* The wild-type amino acid was not conserved at position 148 of APE1 protein and another residue type was observed more often at this position in other homologous sequences. This means that other homologous proteins exist with that other residue type more often than with the wild-type residue in the protein sequence. Therefore, the mutation is possibly damaging.

*Amino Acid Properties.* The wild and mutant type amino acids differ in size, where the mutant residue was found to be bigger, which may lead to altered structure.

### 3.6. MD Simulations of the Wild-Type and Mutant APE1

**Figure 1:**
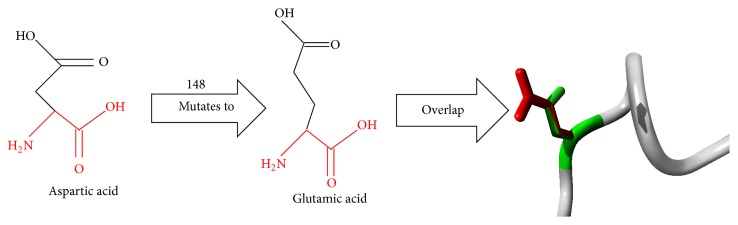
Schematic structure of the wild-type and mutant (Asp148Glu) amino acid.

The 3D structure of APE1 (PDB ID: 2O3H) was already available from the protein database. This structure was used to examine the structural and functional effects of Asp148Glu substitutions in APE1. The structures of the wild-type amino acid aspartate and the risk conferring mutant amino acid glutamate were studied (Figure 1). The backbone was the same for the wild-type and the variant structures (shown in red color), whereas the side chain which was unique for wild and mutant type is shown in black color (Figure 1). Each amino acid depending on its side chain has its own specific size, charge, and hydrophobicity values. The mutant residue (Glu) due to the presence of an additional methylene group was larger in size than the wild-type (Asp) residue. When compared with other homologous protein sequences, the presence of the mutant glutamate reside at its respective position was not found to be conserved and hence could alter the structure and may have deleterious effect on APE1 function.

**Figure 2:**
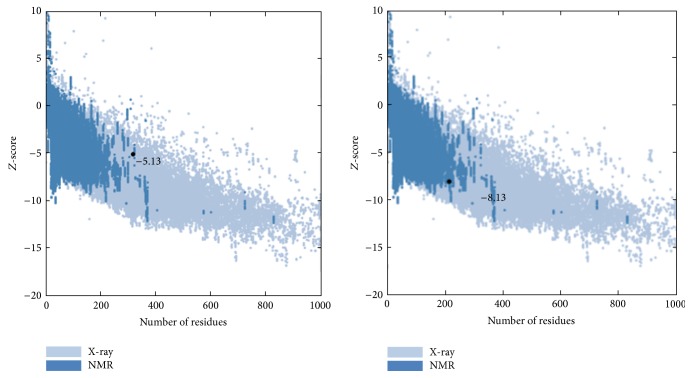
Stability change of the mutant calculated by ProSA server.

Substitution of Asp148 with 148Glu resulted in a slight worsening of ProSA-web *Z*-score, from −5.13 to −8.13 (Figure 2). The total energy deviation was −3 which may have a very unfavorable effect on the APE1 protein structure and function.

**Figure 3:**
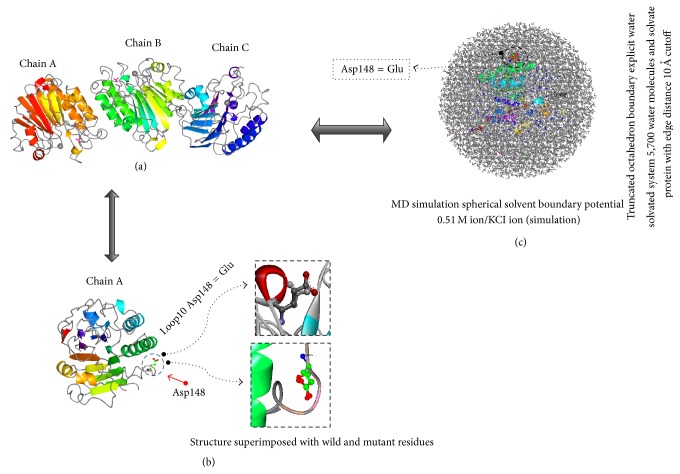
(a) Ribbon diagram of APE1 protein showing all the three side chains A, B, and C. (b) APE1 structure superimposed with wild-type and mutant residue with mutation Asp148Glu enlarged. (c) Molecular dynamics (MD) simulation showing truncated octahedron boundary explicit water solvated and hydrogen atoms. The visual inspection also allows identifying the side chain of Glu residues involved in hydrogen bonding with the surrounding molecules.

Molecular dynamics simulations were carried out using the APE1 structural information from the PDB database. The amino acid sequence and the open reading frame of the APE1 were submitted to I-TASSER program and, of the best five models that were generated, 2O3H was selected based on the *C*-score (−0.92), TM-score (0.60 ± 0.14), RMSD (8.4 ± 4.5 Å), number of decoys (4094), and cluster density (0.0756). The human APE1 (PDB ID: 2O3H) has 285 amino acid residues with three side chains (A, B, and C) (Figure 3(a)). The predicted structure with altered APE1 variant Asp148Glu was studied using Discovery Studio 2.5 and compared with the native structure (Figure 3(b)). The target amino acid at position 148 of APE1 protein was mutated from Asp to Glu and selected for the lowest energy rotamer conformations. The lowest potential energy state was achieved by atomic position arrangements using Steepest Descent (SD) energy minimization protocol for 200 steps and all water molecules were subsequently removed from the resulting structure. The Particle Mesh Ewald summation method was used for the estimation of the electrostatic energy with a distance cutoff of 10 Å. Similar procedure was followed for wild-type APE1 (PDB ID: 2O3H) structure to relax the crystal packing force to compare it with the mutant structure. Consequent to salvation, the resultant solvate showed successful accumulation of solvent around the predicted structure. The octahedral shapes of water box fitted fully to solvate the APE1 protein molecules with an edge distance of 10.0 Å (Figure 3(c)). The wild and the mutant structures were superimposed to detect the effect of structural changes due to the mutation (Figure 3(b)). The structural and functional studies suggest that the variant allele (Asp148Glu) was localized in APE1 binding region; hence the mutation may play a significant role by altering its binding efficacy to its substrate and thus affecting the structural and functional properties of protein.

## 4. Discussion and Conclusions

The apurinic/apyrimidinic endonuclease (*APE*),* APE1*, is involved in the BER pathway [33]. Gene encoding APE1 has five exons with a 2.21 kb coverage on chromosome 14 (14q11.2–q12); when hydrolysed at the 3′ end it blocks DNA oxidisation, thus producing 3′-hydroxyl termini which is required for DNA repair during single- or double-strand breaks [34, 35]. Imbalances in this tightly regulated process due to SNP may cause insufficient DNA repair mechanism and accumulate DNA breaks. In the present study we examined the role of* APE1* variant Asp148Glu and breast cancer risk in Saudi females. The results showed that Asp148Glu variation may increase the risk of breast cancer by approximately 3.5-fold in Saudi patients (Table 2). Furthermore, the results also indicate that the Asp148Glu polymorphism was also associated with increased risk of breast cancer among subgroups of older subjects (>48 years), in ER positive group as well as ER negative group (Tables 3 and 4). It is possible that the older individuals who showed higher risk association with breast cancer were more likely due to aging rather than direct genetic effects. It is more plausible that alteration in the* APE1* gene may be more influential in early onset of breast cancer; however such an association was not observed in our younger group of patients (age ≤ 48 years) probably due to small sample size.

This is the first report that deals with the APE1 variation Asp148Glu which significantly contributes to breast cancer susceptibility in Saudi females and suggests the importance of APE1 in breast carcinogenesis. The elevated risk of breast cancer in subjects with the APE1 alteration (Asp148Glu) can be attributed to the reduced APE1 activity in the DNA base excision repair pathway. Recent meta-analysis study [36] suggests that Asian populations are at higher risk of developing cancer than the non-Asian populations with APE1 Asp148Glu variant. Our results are in agreement with this observation and confirm that the Glu residue at position 148 of the APE1 confers significantly higher risk of breast cancer in Saudi females.

Saudi Arabian population has various tribes settled in different provinces for decades and these are usually recognized by their family names. The families residing in various provinces have been clustered based on their origin [32, 37]. In the present study, the genotype and allele frequencies of rs1130409 (Asp148Glu) in a central region population of Saudi Arabia were observed and compared with various populations of HapMap database. The results showed that the allelic frequencies for rs1130409 (Asp148Glu) were significantly different in the Saudi population compared to GIH, YRI, and MKK populations of HapMap database (Table 5). However, Chinese, Japanese, Italian, and northwestern European populations showed no significant difference in allelic frequencies for rs1130409 variant compared to the Saudi central region population. Hence, examining the SNP variant in other populations probably will not yield similar results, although APE1 (Asp148Glu) has previously been reported to be associated with breast cancer risk in Asian and European populations based on meta-analysis results.

We also evaluated the effect of Asp148Glu mutation on APE1 protein structure. Molecular dynamics methods using simulations in explicit solvent conditions were applied for investigating the wild and mutant amino acids and variation in APE1 protein dynamics and stability due to Asp148Glu variation. The energy minimization studies of the wild-type protein (Asp148) and the mutant type (148Glu) structures revealed that there was energy deviation due to Asp148Glu mutation (−3 kcal/mol). The mutant APE1 (Asp148Glu) structure stability based on thermodynamic changes was also detected using linear mixture of statistical potentials. Protein stability estimation using PoPMusic and CUPSAT revealed that variant Asp148Glu caused structural destabilizing effects on the APE1 protein structure. Structural and functional analysis of the wild-type and variant APE1 revealed numerous multimer contacts including the one associated with nuclease (GO: 0004518) and hydrolase activity (GO: 0016787). APE1 variant Asp148Glu was present in an interpro domain exonuclease/endonuclease/phosphatase (IPR005135) which is responsible for the main activity of the protein; therefore any mutation in this region may affect the function of the protein.

Along with DNA repair activity, APE1 has another major function, which is also known as the redox effector factor 1 (Ref-1) [11]. APE1/Ref-1 reductively activates transcription factors including c-Jun, activator protein-1 (AP-1), nuclear factor kappa B (NF-*κ*B), the tumor-suppressor protein p53, hypoxia-inducible factor 1a (HIF-1a), and paired box gene 8, which are involved in various cellular processes such as cell survival, growth signaling, and inflammatory pathways [38–41]. APE1 was also identified as a direct trans-acting factor for repressing genes by binding to the negative calcium-response element in their promoters. APE1/Ref-1 dysregulation has been reported to be associated with several diseases such as neurodegenerative [42] and cardiovascular diseases [43] and with various human cancers [44, 45]. APE1/Ref-1 may have a role in cancer progression via its ability to increase DNA repair and antiapoptotic, inflammatory, and growth-promoting activities [46]. APE1 gene polymorphisms may lead to amino acid substitutions, which may result in alterations of the functions of APE1/Ref-1.146. Our results support most of the previous studies which stated that Asp148Glu (T/G, codon 148, exon 5, and Asp to Glu) has a role in cancer development and progression. APE1 Asp148Glu mutation is reported to be found among different populations with high frequency and has been associated with various tumors [47–49].

Overall our study has several key findings based on genetic and computational methods to implicate APE1 in the development of breast cancer. The strength of this study is that cancer cases and normal control samples were collected from the central region of Saudi Arabia and errors in genotyping were evaded by replicating select samples for random confirmation of the results. Limitations of these association analyses include the fact that the breast cancer cases were stratified for certain variables such as age at cancer diagnosis and ER status to assess its possible effect; however, the sample size is small and limited to the central region of Saudi population. Hence, in future studies the present data should be validated with larger number of samples as well as in other ethnic groups living in Saudi Arabia.

In conclusion, this is the first study showing an association between the* APE1* Asp148Glu genotypes and increased risk of breast cancer in Saudi patients. Genotyping and* in silico* prediction based on MD simulation results suggest that the* APE1* Asp148Glu variant may alter the BER pathway activity, hence probably contributing to breast carcinogenesis as its dysfunction may play a major role in the development of breast carcinoma. Additional detailed functional as well as association studies with larger sample size are needed to elucidate the role of APE1 polymorphism and associated breast cancer risk in Saudi population.

## Figures and Tables

**Table 1:** Clinical characteristics of study subjects.

Variable	Character	Number of samples
Age (years) Median age (48 ± 8.2^*∗*^)	<48/>48	47/53
Estrogen receptor	ER+/ER−	55/45
Progesterone receptor	PR+/PR−	56/44
HER status	HER+/HER−	40/57

^*∗*^Median age with standard deviation.

**Table 2:** Genotype distribution of APE1 gene polymorphism in breast cancer cases and controls.

Genotype	Cases	Controls	OR	95% CI	*χ* ^2^	*p* value
rs1130409						
Asp/Asp	12 (0.12)	27 (0.27)	Ref			
Asp/Glu	45 (0.45)	46 (0.46)	2.201	0.994–4.872	3.87	0.04917
Glu/Glu	43 (0.43)	27 (0.27)	3.583	1.558–8.243	9.42	0.00215
Asp/Glu + Glu/Glu	88 (0.88)	73 (0.73)	2.712	1.284–5.727	7.17	0.00743
Asp	69 (0.345)	100 (0.5)	Ref			
Glu	131 (0.65)	100 (0.5)	1.899	1.270–2.839	9.85	0.00170

**Table 3:** Genotype frequencies of APE1 gene polymorphism in below 48 and above 48 years of age of breast cancer cases and controls.

Genotype	Cases	Controls	OR	95% CI	*χ* ^2^	*p* value
rs1130409	Below 48					
Asp/Asp	7 (0.148)	16 (0.275)	Ref			
Asp/Glu	24 (0.510)	26 (0.448)	2.110	0.740–6.013	1.99	0.15841
Glu/Glu	16 (0.340)	16 (0.275)	2.286	0.741–7.051	2.11	0.14678
Asp/Glu + Glu/Glu	40 (0.851)	42 (0.724)	2.177	0.810–5.847	2.44	0.11790
Asp	38 (0.404)	58 (0.5)	Ref			
Glu	56 (0.595)	58 (0.5)	1.474	0.851–2.553	1.92	0.16607
rs1130409	Above 48					
Asp/Asp	5 (0.094)	11 (0.261)	Ref			
Asp/Glu	21 (0.396)	20 (0.476)	2.310	0.68–7.84	1.85	0.17378
Glu/Glu	27 (0.509)	11 (0.261)	5.400	1.52–19.20	7.39	0.00656
Asp/Glu + Glu/Glu	48 (0.905)	31 (0.738)	3.406	1.08–10.75	4.70	0.03021
Asp	31 (0.292)	42 (0.5)	Ref			
Glu	75 (0.707)	42 (0.5)	2.419	1.33–4.40	8.53	0.00349

**Table 4:** Genotype frequencies of APE1 gene polymorphism in breast cancer cases ER.

Genotype	Cases	Controls	OR	95% CI	*χ* ^2^	*p* value
rs1130409	**ER +ve**					
Asp/Asp	7 (0.127)	27 (0.27)	Ref			
Asp/Glu	23 (0.418)	46 (0.46)	1.929	0.731–5.089	1.79	0.18065
Glu/Glu	25 (0.454)	27 (0.27)	3.571	1.322–9.645	6.65	0.00992
Asp/Glu + Glu/Glu	48 (0.872)	73 (0.73)	2.536	1.023–6.286	4.22	0.03992
Asp	37 (0.336)	100 (0.5)	Ref			
Glu	73 (0.663)	100 (0.5)	1.973	1.217–3.198	7.71	0.00551
rs1130409	**ER −ve**					
Asp/Asp	5 (0.111)	27 (0.27)	Ref			
Asp/Glu	22 (0.488)	46 (0.46)	2.583	0.876–7.613	3.09	0.07881
Glu/Glu	18 (0.4)	27 (0.27)	3.600	1.168–11.09	5.30	0.02127
Asp/Glu + Glu/Glu	40 (0.888)	73 (0.73)	2.959	1.057–8.281	4.56	0.03281
Asp	32 (0.355)	100 (0.5)	Ref			
Glu	58 (0.644)	100 (0.5)	1.812	1.085–3.027	5.22	0.02230

**Table 5:** Allele and genotype frequencies of APE1 rs1130409 (Asp148Glu) in central Saudi Arabia and other populations.

Population	Genotype freq. (number)			Allele frequency		Pairwise *χ* ^2^ test value between CRS & other populations	*p* value
	Asp/Asp freq. (number)	Asp/Glu freq. (number)	Glu/Glu freq. (number)	Wild type Asp	Variant Glu		
CEU (*n* = 226)	0.319	0.407	0.274	0.52	0.48	**0.13**	0.71
CHB (*n* = 84)	0.214	0.476	0.310	0.45	0.55	**0.41**	0.51
JPT (*n* = 170)	0.094	0.494	0.412	0.34	0.66	**6.61**	0.01
GIH (*n* = 176)	0.114	0.341	0.545	0.28	0.72	**12.86**	0.0003^*∗*^
YRI (*n* = 226)	0.133	0.381	0.487	0.32	0.68	**9.24**	0.002^*∗*^
MKK (*n* = 286)	0.098	0.524	0.378	0.36	0.64	**6.05**	0.01
TSI (*n* = 174)	0.230	0.448	0.322	0.45	0.55	**0.53**	0.46
CRS (*n* = 100)	0.27	0.46	0.27	0.50	0.50	**Ref**	—

Population descriptions:

CEU: Utah residents with Northern and Western European ancestry from the CEPH collection.

CHB: Han Chinese in Beijing, China.

JPT: Japanese in Tokyo, Japan.

GIH: Gujarati Indians in Houston, Texas.

YRI: Yoruba in Ibadan, Nigeria.

MKK: Maasai in Kinyawa, Kenya.

TSI: Toscans in Italy.

CRS: Saudi population residing in Riyadh region of central Saudi Arabia.

^*∗*^ *p* values significant after Bonferroni correction.

## Abbreviations

APE1: Apurinic/apyrimidinic endonuclease 1
MD simulation: Molecular dynamics simulation
PoPMuSiC: Prediction of Protein Mutant Stability Changes
CUPSAT: Cologne University Protein Stability Analysis Tool
HOPE: Have yOur Protein Explained.
